# Changes in Brain Monoamines Underlie Behavioural Disruptions after Zebrafish Diet Exposure to Polycyclic Aromatic Hydrocarbons Environmental Mixtures

**DOI:** 10.3390/ijms18030560

**Published:** 2017-03-04

**Authors:** Caroline Vignet, Verena M. Trenkel, Annick Vouillarmet, Giampiero Bricca, Marie-Laure Bégout, Xavier Cousin

**Affiliations:** 1 Laboratoire Ressources Halieutiques, Ifremer, Place Gaby Coll, 17137 L’Houmeau, France; vignet.caroline@gmail.com (C.V.); mlbegout@ifremer.fr (M.-L.B.); 2 Unité Écologie et Modèles pour l’Halieutique, Ifremer, B.P. 21105, 44311 Nantes CEDEX 03, France; Verena.Trenkel@ifremer.fr; 3 Génomique Fonctionnelle de l'Hypertension Artérielle, EA 4173, University Lyon 1, 8 Avenue Rockefeller, 69373 Lyon CEDEX 08, France; annick.vouillarmet@univ-lyon1.fr; 4 Laboratoire Adaptation et Adaptabilités des Animaux et des Systèmes, UMR MARBEC, Ifremer, Route de Maguelone, 34250 Palavas, France; 5 GABI, INRA, AgroParisTech, Université Paris-Saclay, 78350 Jouy-en-Josas, France

**Keywords:** monoamine, polycyclic aromatic hydrocarbons, behaviour, anxiety, swimming activity, zebrafish

## Abstract

Zebrafish were exposed through diet to two environmentally relevant polycyclic aromatic hydrocarbons (PAHs) mixtures of contrasted compositions, one of pyrolytic (PY) origin and one from light crude oil (LO). Monoamine concentrations were quantified in the brains of the fish after six month of exposure. A significant decrease in noradrenaline (NA) was observed in fish exposed to both mixtures, while a decrease in serotonin (5HT) and dopamine (DA) was observed only in LO-exposed fish. A decrease in metabolites of 5HT and DA was observed in fish exposed to both mixtures. Several behavioural disruptions were observed that depended on mixtures, and parallels were made with changes in monoamine concentrations. Indeed, we observed an increase in anxiety in fish exposed to both mixtures, which could be related to the decrease in 5HT and/or NA, while disruptions of daily activity rhythms were observed in LO fish, which could be related to the decrease in DA. Taken together, these results showed that (i) chronic exposures to PAHs mixtures disrupted brain monoamine contents, which could underlie behavioural disruptions, and that (ii) the biological responses depended on mixture compositions.

## 1. Introduction

Monoamine neurotransmitters are implicated in the regulation of a large number of processes such as motor control, social behaviour, cognition, sleep, appetite, and anxiety in vertebrates [1]. In zebrafish, serotonin (5HT) and dopamine (DA) are the two most studied monoamines. The amenability of zebrafish early life stages (embryo and larvae) has allowed refined analyses of serotonin and dopamine neuropharmacology and behavioural correlates, while studies in later life stages (juveniles and adults) are scarcer. It has been demonstrated that the two neurotransmitters play a similar role to that observed in other vertebrates [2,3,4,5,6,7,8].

In wild animals, disruption of key behaviours such as social, exploration, or mating behaviours may result in a dramatic decrease in individual fitness with consequences for population sustainability. The study of the consequences of pollutants exposures on behaviour has recently increased, especially for fish, in parallel to the expansion of the use of behaviour as an efficient indicator for neurotoxicity in pharmacology, toxicology, and ecotoxicology. With respect to this, we and others have shown that the exposure of fishes to pollutants using environmentally relevant exposure conditions can induce behavioural disruptions at various developmental stages (e.g., [9,10,11,12,13,14,15,16,17,18]). Other studies have shown that exposure to pollutants could modify monoamine neurotransmitter concentrations (e.g., [19,20,21,22,23]). However, the simultaneous study of the consequences of fish pollutant exposures on behaviour and neurotransmitter concentrations is much more limited and has been shown for example after exposure to chlorpyrifos [24,25] and tributyltin [26].

Polycyclic aromatic hydrocarbons (PAHs) are a large family of organic pollutants emitted as complex mixtures into the environment as a consequence of human activities [27,28,29]. Among PAHs, one can find low molecular weight (LMW) PAHs, such as phenanthrene and fluoranthene that have two to three aromatic rings, and high molecular weight (HMW) PAHs, such as pyrene and benzo[a]pyrene (BaP), which have four or more aromatic rings. In addition to these parent compounds, one can also find substituted PAHs including alkylated, nitrogenated, halogenated, and hydroxylated. Depending on their origin, the composition of PAHs mixtures varies as well as their potential mode of action. Pyrolytic mixtures result from the combustion of organic matter including fossil fuel; they enter aquatic environments through the deposition of atmospheric emissions directly on water or on the ground followed by soil runoff. Pyrolytic mixtures of PAHs contain a high proportion of HMW PAHs. Petrogenic mixtures originate from oil and the degradation of oil-based products and enter the aquatic environment due to harbour activity or as a consequence of oil spills. Petrogenic mixtures contain high proportions of LMW and alkylated PAHs. Historically, environmental monitoring programs have used a subset of 16 PAHs established by the U.S. Environmental Protection Agency (EPA; see construction of the 16 U.S.-EPA PAHs list in [30]). Since then, it has been shown that this subset lead to an underestimation of actual PAHs concentrations in environmental samples and their toxicity and the list has been debated [31]. However 16 U.S.-EPA PAHs list is still frequently used in monitoring programs.

As for other organic pollutants, PAHs have been shown to induce changes in neurotransmitters concentration in fish brain [20,21,22,23] and behavioural changes [15,16,18,32]. However, individual PAHs (mainly BaP) have been used in all these cases while the use of PAHs mixtures better representative of environmental situations is scarce [9,17].

In this work, we performed long-term chronic dietary exposures of zebrafish to two PAH mixtures [33]. PY aromatic fraction was extracted from Seine estuary sediment and was representative of pyrolytic PAHs while the other aromatic fraction, representative of petrogenic PAH mixtures, was extracted from Light crude oil and named LO. In both cases, the targeted concentrations for the 16 U.S.-EPA PAHs were representative of those observed in wild molluscs, considered as possible prey for fish, sampled in polluted areas at 15 μg·g^−1^. Actual concentrations were 15.3 ± 4.2 μg·g^−1^ for PY and 4.1 ± 0.6 μg·g^−1^ for LO diets for the 16 U.S.-EPA PAHs while total PAH concentrations were 18.2 ± 5.0 and 19.6 ± 1.9 μg·g^−1^ respectively. The behavioural responses of zebrafish were monitored for juveniles and adults following [9] with a particular focus on structure of diurnal activity. To further explore possible mechanisms of action, the concentration of monoamines and their metabolites were measured in the brain after the same exposure duration.

## 2. Results and Discussion

Zebrafish exposures started from the first meal and lasted until behavioural experiments were performed and brain sampled. The pyrolytic mixture (PY) contained high levels of HMW PAHs and almost no LMW PAHs. The petrogenic mixture (LO) contained low level of HMW PAHs and high level of LMW PAHs. In addition LO contained high proportion of alkylated LMW PAHs [33]. Actual concentrations measured in diet after spiking was 15.3 ± 4.2 µg of PAHs per g of food for PY and 4.1 ± 0.6 µg·g^−1^ for LO according to concentration of the 16 U.S.-EPA indicator PAHs corresponding to a spiking efficiency of 100% and 56% for PY and LO mixtures respectively (see Supplementary Information 1 for detailed PAHs concentrations). Therefore PAHs concentrations in diets effectively corresponded to concentrations measured in flesh of molluscs sampled in contaminated areas [34] underlying the environmental relevance of exposure conditions used. Monitoring of PAH metabolites in larvae sampled at 15 days post fertilisation (dpf), after 10 days of exposure to PY and LO diets, confirmed actual contamination of fish [33]. In addition to PAHs and other organic compounds, Oissel native sediment sampled also contained heavy metals which were not retained in the aromatic fractions [35]. Among organics, polychlorinated biphenyls and lindane have been detected but at concentrations in sediment more than two order of magnitude lower than PAHs for polychlorinated biphenyls and more than four order of magnitude lower for lindane. So even if we cannot rule out the effect of these compounds on behaviour, this is very unlikely.

**Figure 1:**
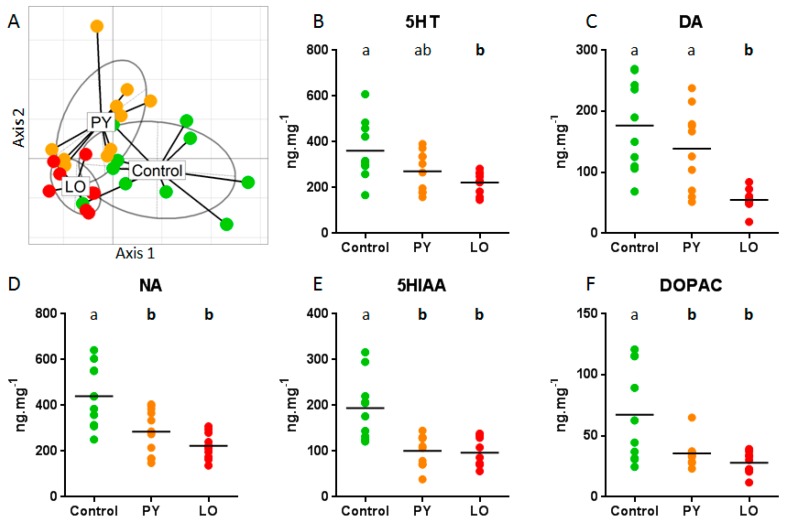
Analysis of monoamine brain content. (**A**) principal component analysis (PCA) global analysis of brain monoamine concentrations clearly identify pyrolytic (PY) and light crude oil (LO) as different from Control group. PCA axis 1 contributed to 82.1% of variability and PCA axis 2 for an additional 8.8%; (**B**–**F**) Detailed analysis of brain monoamines and metabolites concentrations in PY, LO and Control fish. Each dot represents results from an individual fish. Letters indicate significant differences at *p* < 0.05 between diets.

Noradrenaline (NA), dopamine (DA) as well as 3,4-dihydroxyphenylaceticacid (DOPAC; one metabolite of DA), serotonin (5HT), and 5-hydroxyindole-3-aceticacid (5HIAA; one metabolite of 5HT) were measured in adult whole brains. No significant difference was observed between sexes for any analysed compounds whatever the diet (Supplementary information 2). Further statistical analyses were therefore performed considering both sexes together. Considering monoamines concentrations, using a principal component analysis, we found that more than 90% of total variability was explained by the first two axes with principal component analysis (PCA) axis 1 explaining 82.1% of variability (Figure 1A). This indicates that the five monoamines were highly correlated. More than 40% of the variability in the first two axes could be explained by diet treatment indicating a significant effect of zebrafish exposure on brain monoamine concentrations (*p* < 0.001). The remaining variability was linked to individual variability independently of the diet. Inspection of between-treatment PCA results indicated that differentiation between exposed (PY and LO) and Control fish was along PCA axis 1, while differences between PY and LO and to a lesser extent Control group was along PCA axis 2 (Figure 1A). All monoamines similarly contributed to PCA axis 1 [component loadings 0.39–0.49] with a major contribution of NA and 5HIAA (see Supplementary Information 3 for detailed respective contributions). Along axis 2 the major contributors was DA (+0.81) while 5HIAA (−0.54) and DOPAC (−0.22) contributed negatively to this axis.

A non-parametric approximation to multivariate ANOVA followed by pairwise comparisons was performed to analyse in further details respective changes in monoamine and metabolites concentrations (Figure 1B–F and Supplementary Information 3). Again, the treatment effect was highly significant (*p* < 0.001). For catecholamines, a significant decrease in NA concentration was observed for both contaminated diets compared to Control, while in the case of DA, only fish exposed to LO diet showed a reduced concentration compared to Control. The ratio DOPAC/DA increased in brain of fish exposed to LO diet indicative of an increase in DA metabolism. Indeed, compared to Control, DA was significantly lower in LO treated individuals but not those treated with PY while DOPAC was lower in both. For tryptamines, a significant decrease in 5HT concentration was observed in brain of fish exposed to LO diet compared to Control while for PY diet, concentration was intermediate. A significant decrease in 5HT metabolite, 5HIAA, was observed in brain of fish exposed to both contaminated diet. The ratio 5HIAA/5HT, decreased in brain of PY fish indicative of a decrease in 5HT metabolism. In the case of LO there was a trend to such a reduction in pairwise analysis of the ratio between LO and Control. These findings clearly suggested neurotoxicity after exposure to PAHs mixtures. They also indicated that effects were different depending on the mixture used and indeed DA and 5HT concentrations in PY brains were not different from Control albeit a trend to a decrease in 5HT was observed.

In previous studies only acute exposures to individual compounds (BaP, β-naphthoflavone or naphthalene) have been performed and therefore comparison with previous work is not straightforward. However, it has been shown that, in rat, acute exposure to BaP (intra-peritoneal (ip) injection; 50 mg·kg^−1^; maximum duration 96 h) decreased DA in forebrain and NA in forebrain and midbrain [36]. In another study performed with BaP in rat (three times i.p. injection; 10 mg·kg^−1^; ≤24 h), variations of DA concentrations depended on the brain regions considered with an increase in the hippocampus and a decrease in the caudate putamen while the ratio DOPAC/DA was increased in the caudate putamen and the nucleus accumbens [37]. In rainbow trout, acute exposure to BaP (i.p. injection; 10 mg·kg^−1^; ≤72 h) produced a decrease in 5HT in several regions of the brain [21] while exposure to naphthalene (i.p. injection; 10–50 mg kg^−1^; ≤120 h) produced an increase or a decrease in DA and 5HT depending on brain regions [23]. In these reports, monoamine concentrations remained unchanged in other brain areas. Recently, it has also been reported that exposure of zebrafish during more than 7 months, starting with embryos, to low BaP concentrations (waterborne exposure; effects seen starting at 0.05–0.5 nM (12.6−126 ng·L^−1^) resulted in decrease in both DA and DOPAC, induced neurodegeneration and behavioural changes in adults [32]. Taken together, our results are in agreement with previous works which reported disruptions in brain monoamine concentrations upon exposures to BaP. The fact that in our case, all changes corresponded to a decrease in whole brain could be due to the duration of the exposure leading to permanent or chronic disruptions in monoamine pathways.

**Figure 2:**
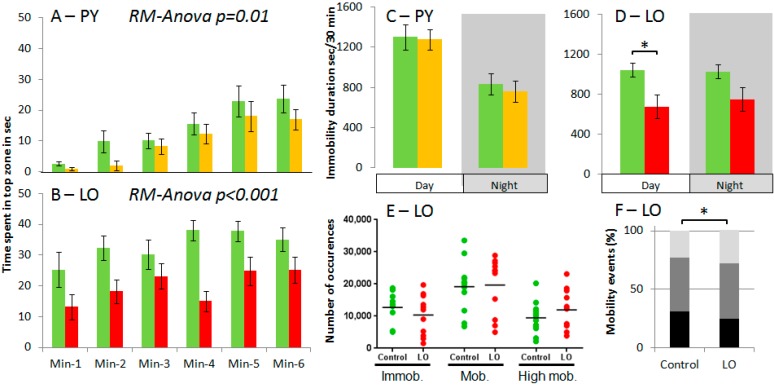
Analysis of behavioural traits. (**A**,**B**) Mean time spent in top zone as evaluation of anxiety using novel-tank paradigm; (**C**,**D**) Immobility duration during day and night in a 24 h-swimming test; (**E**) Number of events in each mobility class for individual fish and mean; and (**F**) Relative proportion of Immobility (black), mobility (dark grey) and high mobility (light grey) events during day period in a 24 h-swimming test. *n* = 12 individuals; values are mean ± standard error of the mean; except in **E** (individual values and means) and **F** (means). In **A** and **B** since RM-Anova takes all time points into account the *p*-value is indicated in the figure, otherwise * indicates significant difference at *p* < 0.05 for PAHs diets compared to the Control.

The novel-tank test has been widely used to evaluate anxiety level. When introduced into the novel tank, fish initially occupy the bottom of the tank (homebase behaviour), then the time spent in top zone of the tank progressively increases. Anxious fish spend less time in top zone than non-anxious fish [38]. Compared to Control fish, PY and LO exposed fish spent significantly less time in top zone (respectively *p* = 0.01 and *p* < 0.001; Figure 2A,B). This is indicative of an increase in anxiety level and has been observed both at 2 and 6 month of age [9]. This lower increase could also be due to other effects not related to anxiety. For example, distance travelled by a fish could be reduced along with its size, which was reduced for both PY and LO fish compared to Control fish [33]. However, distance travelled is not modified for PY or LO fish [9], indicating that the reduced time in top zone of the tank during the challenge cannot be explained by a reduced swimming ability. Another possible mechanisms could be metabolism deficiency. However, the absence of changes in distance travelled again does not support this hypothesis. In addition, some metabolic traits (standard metabolic rate, active metabolic rate or aerobic metabolic scope) have been monitored in fish exposed to the same PY fraction as in present work and concluded to the absence of metabolic effect [39,40]. Unfortunately LO fish have not been tested. This suggests that the lower time spent in the top zone of the tank could be related to an increase in anxiety. This more anxious state could be related to the joint decrease in 5HT and/or NA in brain of both PY and LO fish since both 5HT and NA reuptake inhibitors led to an increase in anxiety in zebrafish [3,41,42].

Spontaneous swimming activity was monitored over 24 h (Figure 2C,D) and activity of exposed fish was statistically undistinguishable from Control fish during night period. During day period, distances travelled by fish were similar whatever the diet but fish exposed to LO diet displayed a significant decrease in immobility total duration (*p* < 0.001). This indicated an increase in overall diurnal activity [9]. In order to characterise in more details this changes in diurnal activity, we compared the number of occurrences according to the mobility state for LO fish (Figure 2E). The comparison of each mobility category revealed a change in their relative proportions (χ-square = (6216, 2); *p* < 0.0001; Figure 2F). These changes were due do a decrease in immobility occurrence and an increase in high mobility occurrence for LO fish compared to Control fish and this is indicative of a decrease in the resting time for LO fish. This is supported at the individual level by the correlation analysis of the number of occurrences per category which were highly correlated for Control fish (Spearman rank order correlations in the [0.62–0.85] range, see Supplementary Information 4) while in the case of LO fish only the occurrences of mobile and immobile categories were correlated. This suggests a modification of diurnal activity structure. This may be related to a disruption of melatonin expression pattern during day-period as observed in trout after exposure to naphthalene [43]. Alternatively, this could be related to the decrease in DA, observed only for LO fish. Indeed it has recently been shown that a decrease in DA in mouse brain led to a shortening of ultradian activity period (higher fractionation of activity, [44]). Our results are also in agreement with behavioural changes observed in adult zebrafish after seven months of exposure to low BaP concentrations (waterborne exposure; effects seen starting at 0.05–0.5 nM (12.6–126 ng·L^−1^)). More precisely, when tested for spontaneous activity, exposed fish display a dose-dependent decrease in activity (distance travelled and swimming speed). Exposed fish have also been shown to have decreasing learning abilities after chronic exposure [32] or long after early exposure [18].

These results indicated that exposure to environmentally relevant PAH mixtures could disrupt behaviours that may have detrimental consequences in terms of the performance of the exposed fish, their ability to survive and to explore their environment, and hence their ability to contribute to the next generation. They also showed that behavioural defects (activity rhythms disruption and/or increase in anxiety) may differ depending on the composition of PAH mixtures, pointing at a major role for LMW and methylated PAHs. Exposures performed in this article included early life stages (larval stages); it is therefore possible that effects may be related to early disruptions occurring during nervous system development and maturation. In regards to this point, it should be mentioned that early (embryo and larvae in fish and perinatal in mammals) exposure to PAHs has already been shown to induce behavioural disruptions [45,46,47,48,49,50].

## 3. Material and Methods

### 3.1. Diet Preparation

Aromatic fractions, diet preparation, and exposure have been previously described in details [33]. Briefly, aromatic fractions were prepared after extraction from (1) sediments collected from a polluted site in the Seine Estuary (Oissel, France; PY mixture) with Accelerated Solvent Extraction (ASE300, Dionex, Sunnyvale, CA, USA) using dichloromethane as solvent and from (2) Arabian Light crude oil (LO mixture) after dissolution in pentane to induce asphaltene precipitation. Both extracts were then purified using alumina columns and eluted with dichloromethane. After another reconcentration step, aliphatic fractions obtained after elution on silica columns with pentane were discarded and aromatic fractions were obtained using pentane/dichloromethane (65/35, *v*/*v*) as solvents [51,52]. Both extracts were used to spike food pellets (INICIO Plus 0.5, Biomar, France) ground to fish age-appropriate sizes. For each exposure, a control treatment was included, corresponding to the plain food treated as for the spiked food with dichloromethane, the carrier solvent used for PAH spiking. The targeted concentration for spiking, 15 μg·g^−1^, was based on concentrations measured in mussels sampled in polluted areas [34]. After spiking, PAHs were extracted by SPME (Solid-Phase MicroExtraction) and then quantified by GC/MS (Agilent GC 7890A/Agilent MSD 5975C, Agilent Technology, California, CA, USA) along with deuterated standard solutions. Spiking efficiency was calculated based on the targeted and actual concentrations for the 16 U.S.-EPA PAHs and was 100% for PY fraction (15.3 ± 4.2 μg·g^−1^) and 56% for LO fraction (4.1 ± 0.6 μg·g^−1^).

### 3.2. Fish Exposure

This study was conducted with the approval of the French Animal Care Committee under project authorization number CE2012-23. We used the zebrafish wild-type Tuebingen (TU) strain (ZFIN ID: 76-ZDB-GENO-990623-3). Eggs were obtained by random pairwise mating, sorted and distributed in Petri dishes to constitute groups by mixing embryos from at least five spawns. From 5 days post fertilisation (dpf) onward, the fish were fed twice daily with size-adapted spiked pellets and once daily with plain Artemia nauplii. Spiked-food was initially distributed ad libitum and only after the first biometry (between 2 and 3 months of age) food was distributed with a daily ration set at 2% body mass. Exposure with spiked diet was therefore continuous starting at 5 dpf. During the ConPhyPoP project [33], more than 50 replicates were produced and fish used in the present study originated from three replicates.

### 3.3. Behaviour Analysis

Behavioural traits (anxiety and activity) were monitored using appropriate apparatus back lighted with infrared devices and fish swimming behaviour was recorded with an analogue ICD-48E camera (Ikegami; Elvitec, Pertuis, France), equipped with a 2.7–13.5 mm lens (Fujinon; Elvitec, Pertuis, France) and an infrared filter (The Imaging Source; Elvitec, Pertuis, France), linked to a PC with an acquisition card and Ethovision XT software (Noldus, Wageningen, The Netherlands). In all experiments, EthoVision XT software was used for track extraction and analysis. Twelve naive fish were monitored for each trait at 2 and 6 months of age.

Anxiety level was monitored using the Novel tank paradigm [38]. The day before the challenge, fish were transferred into individual 1 L-tanks in the test room. Tests were always performed between 14:00 and 16:00. Fish were transferred to a novel tank (trapezoid 1.5 L tank; Aquatic Habitats) and filmed for 6 min from the side. The dependent variable measured was the time spent in the top half zone of the tank which is inversely proportional to anxiety level.

Spontaneous swimming activity was monitored in 3 L tanks (24.5 × 15 × 13.5 cm, AquaBox^®^ 3, AquaSchwarz, Göttingen, Germany) filled with 1.5 L of system water. Twelve tanks were used simultaneously and were isolated from neighbouring tanks by opaque walls. Fish were placed in the tanks at 17:00 on the day before the experiment, to allow them to acclimate overnight. Video recordings began the next day at 12:30 and lasted 24 h including day (14 h) and night (10 h) periods. Photoperiod was synchronised with the rearing room. The dependent variables measured were the distance travelled and mobility. Mobility was described with three categories: high mobility, mobility, and immobility, and each category has a measure of occurrence and duration (Ethovision XT default settings were used: high mobility corresponds to a change in overlapping of body surface in pixels between two frames of more than 15%, while immobility corresponds to a change of less than 2%; mobility falls in between).

### 3.4. HPLC Analysis of Whole Brain

Zebrafish from both sexes were sacrificed for all three diet treatments at 6 month of age using a lethal dose of benzocaine. Brains were quickly dissected, flash frozen in liquid nitrogen, and stored at −80 °C. The HPLC methods used were those developed for zebrafish [53] using the UHPLC system (Waters Acquity, Chicago, IL, USA). Dopamine, DOPAC (one metabolite of DA), noradrenaline, serotonin and its metabolite 5HIAA were quantified in whole brains from Control (*n* = 10), PY (*n* = 10) and LO (*n* = 9) treatments using their respective standards (Sigma Aldrich, Saint-Quentin Fallavier, France) to identify the peaks on the chromatograph. The concentrations of neurotransmitters and metabolites were expressed as ng per mg of tissue.

### 3.5. Statistical Analysis

To study the impact of contaminated diet on monoamine concentrations we carried out a between-treatment principal component analysis (PCA) [54] for the five biochemistry variables. For this we first carried out a PCA on the five biochemistry variables ignoring treatment. We then modeled the first two axes of this first PCA using treatment as explanatory variable and tested for significant differences between treatments by Monte Carlo simulation (99,999 simulations). Analyses were carried out in the statistical environment R [55] using packages ade4 [56] (functions dudi.pca, bca and randtest) and adegraphics [57].

To further test the treatment effect, we used a non-parametric approximation to a multivariate analysis of variance with the five biochemistry variables as multivariate dependent variables and treatment as explanatory variable [54]. For this, the nonpartest function in the R package npmv was used [58]. *p*-Values were estimated by permutation (1000 permutations). We then pairwise compared mean concentrations between treatments for each biochemistry variable separately. Analyses were carried out in R and using package lsmeans [59].

Other effects were statistically tested using Statistica 9.0 (Statsoft, Tulsa, OK, USA). We first verified data normality and homoscedasticity. Then, before calculating both ANOVA and repeated-measures ANOVA, we tested for the effects of the random factors individual and tank (replicate origin) and the fixed factors sex and treatment on the variable of interest using a generalized linear mixed-model (GLMM) and could not see any effects of the random factors or sex (for details see [9]). Therefore, we simplified our analysis by removing these factors and focusing on treatment (diets) only. To compare our results between treatments we used either ANOVA (high mobility and immobility duration in 24 h-swimming test) or repeated-measures ANOVA (time spent in top zone for novel tank experiment). χ -square test was used to compare the proportion of mobility state occurrences between treatments. Spearman rank order correlations were calculated within treatments for the mobility events of the different categories (mobile, immobile and highly mobile). All statistical analyses were carried out at a 5% level of significance.

## 4. Conclusions

Using long term exposures of zebrafish to environmentally relevant mixtures of PAHs (both in terms of compositions and concentrations), we showed changes in brain monoamines content along with behavioural disruptions. Both PY and LO exposures produced a decrease in 5HT and NA that could be related to the observed increase in anxiety, while the disruption of daily activity rhythm in LO fish could be related to the decrease in DA only observed in those fish. These differences were likely caused by the different compositions of PY and LO mixtures, suggesting both common and distinct pathways and mode of action.

## Supplementary Materials

Supplementary materials can be found at www.mdpi.com/1422-0067/18/3/560/s1.
