# Supplementary material for: Changes in Brain Monoamines Underlie Behavioural Disruptions after Zebrafish Diet Exposure to Polycyclic Aromatic Hydrocarbons Environmental Mixtures
Source: Int J Mol Sci. 2017 Mar 4;18(3):560. doi: 10.3390/ijms18030560 (PMC5372576; doi:10.3390/ijms18030560)

Supplementary information 1 Detailed PAH concentrations for produced diets in nanograms per gram of food (mean ± SD; n=4–7).

	Ring #		16 US-EPA	Control	PY	LO
naphthalene	2	LMW	x	4 ± 2	157 ± 74	1110 ± 472
acenaphthylene	2	LMW	x	1 ± 0	114 ± 23	136 ± 19
acenaphthene	2	LMW	x	11 ± 17	89 ± 24	90 ± 30
fluorene	2	LMW	x	2 ± 1	137 ± 28	677 ± 55
dibenzo[*b,d*]thiophene	2	LMW		1 ± 0	102 ± 26	3489 ± 208
phenanthrene	3	LMW	x	7 ± 4	895 ± 213	1438 ± 86
anthracene	3	LMW	x	1 ± 0	482 ± 165	42 ± 54
fluoranthene	3	LMW	x	2 ± 1	1782 ± 353	15 ± 17
pyrene	4	HMW	x	2 ± 0	1496 ± 311	73 ± 28
benzo[a]anthracene	4	HMW	x	1 ± 0	1671 ± 763	49 ± 28
triphenylene + chrysene	4	HMW	x	1 ± 0	2144 ± 1032	320 ± 62
benzo[*b*]naphto[2,1-*d*]thiophene	4	HMW		5 ± 3	472 ± 230	588 ± 30
benzo[b]fluoranthene+benzo[k]fluoranthene+benzo[j]fluoranthene	4	HMW	x	2 ± 1	2740 ± 674	66 ± 7
benzo[*e*]pyrene	5	HMW		1 ± 0	1084 ± 286	160 ± 10
benzo[a]pyrene	5	HMW	x	0 ± 0	1168 ± 346	17 ± 3
perylene	5	HMW		1 ± 0	390 ± 83	13 ± 1
indeno(1,2,3-cd)pyrene	5	HMW	x	0 ± 0	1188 ± 265	0 ± 0
dibenz(ah)anthracene + dibenz(ac)anthracene	5	HMW	x	2 ± 2	301 ± 106	11 ± 1
benzo[ghi]perylene	6	HMW	x	0 ± 0	893 ± 191	42 ± 12
**Sum 16 US-EPA PAHs**				**30 ± 19**	**15257 ± 4195**	**4086 ± 635**
**Sum parents PAHs**				**34 ± 19**	**17305 ± 4798**	**8335 ± 854**
2-methylnaphthalene	2			5 ± 2	116 ± 39	2982 ± 725
1-methylnaphthalene	2			2 ± 1	62 ± 21	3300 ± 739
**Sum methylnaphthalenes**				**7 ± 3**	**178 ± 60**	**6282 ± 1465**
3-methylphenanthrene	3			2 ± 1	149 ± 31	850 ± 156
2-methylphenanthrene	3			2 ± 1	175 ± 41	915 ± 124
2-methylanthracene	3			1 ± 0	78 ± 18	32 ± 8
9-methylphenanthrene + 1-methylanthracene	3			1 ± 0	165 ± 56	2226 ± 394
1-methylphenanthrene	3			1 ± 0	100 ± 28	956 ± 97
**Sum methylphenanthrenes**				**5 ± 3**	**668 ± 157**	**4957 ± 731**
**Total PAHs**				**55 ± 12**	**18151 ± 4983**	**19574 ± 1945**

Supplementary information 2 Detailed results of monoamines concentrations according to sex of fish. In A) p-values after Mann-Whitney tests to compare concentrations in females and males within each treatment.

A

	Control	PY	LO
5HT	0.39	0.60	0.80
DA	0.09	0.12	0.61
NA	0.09	0.75	0.20
5HIAA	0.39	0.60	1.00
DOPAC	0.29	0.46	1.00

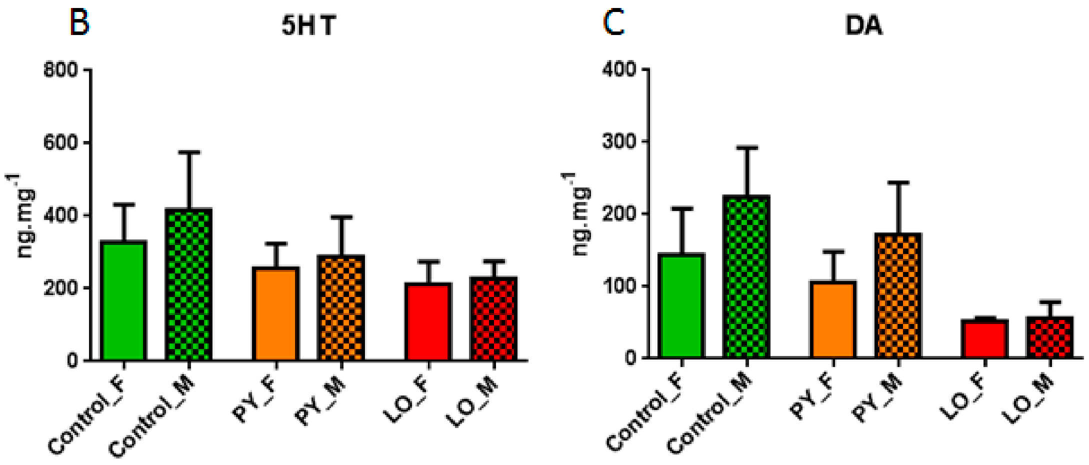

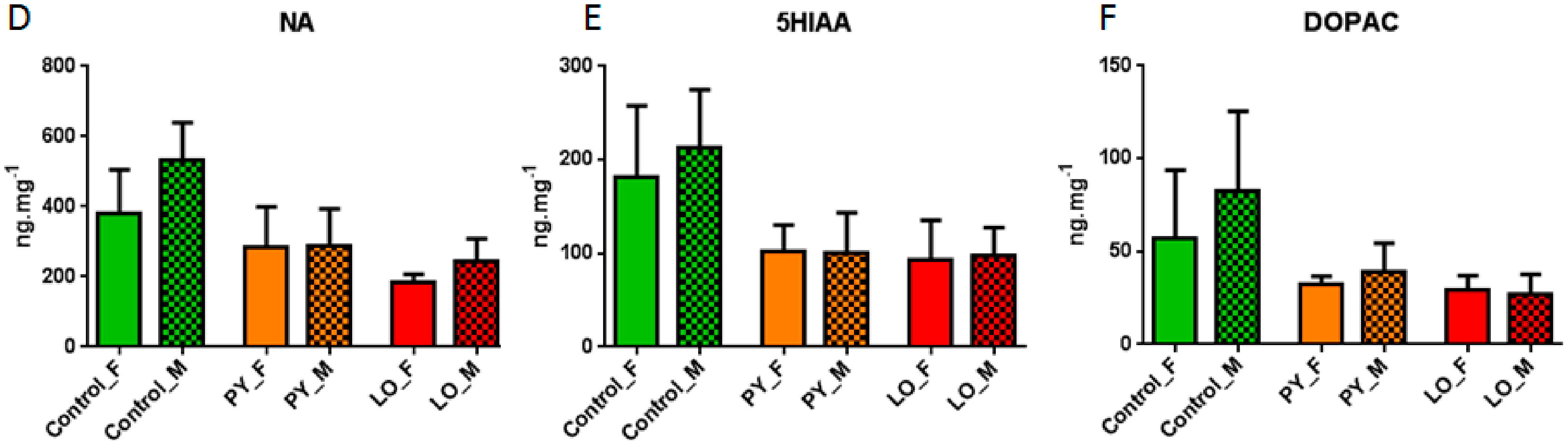

Supplementary information 3 Detailed results of monoamines concentrations PCA.

Projected inertia (%):

Axis 1	Axis 2	Axis 3	Axis 4	Axis 5
82.069	8.838	5.742	1.937	1.415

Contribution of variables to two main axes:

	Axis1	Axis2
DA	0.438	0.808
DOPAC	0.426	-0.216
5HT	0.388	0.072
5HIAA	0.491	-0.540
NA	0.485	-0.051

Monoamine concentrations in brain (ng.mg^{-1} tissue; mean ± SD):

	Control				PY				LO			
DA	176.55	±	18.77	**a**	138.75	±	18.77	**a**	54.89	±	19.79	**b**
DOPAC	67.29	±	7.76	**a**	35.66	±	7.76	**b**	28.00	±	8.18	**b**
5HT	361.90	±	30.34	**a**	271.80	±	30.34	**ab**	222.11	±	31.98	**b**
5HIAA	194.10	±	15.37	**a**	100.95	±	15.37	**b**	96.49	±	16.20	**b**
NA	440.40	±	33.87	**a**	285.95	±	33.87	**b**	222.83	±	35.70	**b**

Letters indicate differences at p<0.05 between diets.

Ratio of metabolites/precursors concentrations (mean ± SD):

	Control				PY				LO			
DOPAC/DA	0.37	±	0.1	**a**	0.53	±	0.12	**a**	0.53	±	0.12	**b**
5HIAA/5HT	0.56	±	0.18	**a**	0.43	±	0.07	**b**	0.43	±	0.07	**ab**

Letters indicate differences at p<0.05 between diets.

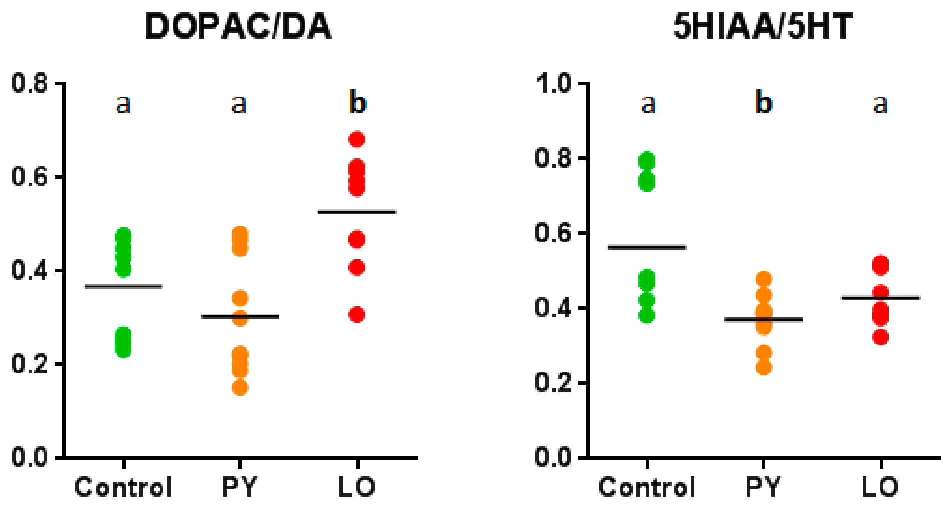

Ratio DOPAC/DA and 5HIAA/5HT. Individual values and means. Letters indicate significant difference at p<0.05 between diets.

Supplementary information 4 Correlation between mobility events according to mobility state. Number in each graph indicates significant Spearman rank order correlations at $p<0.05$.

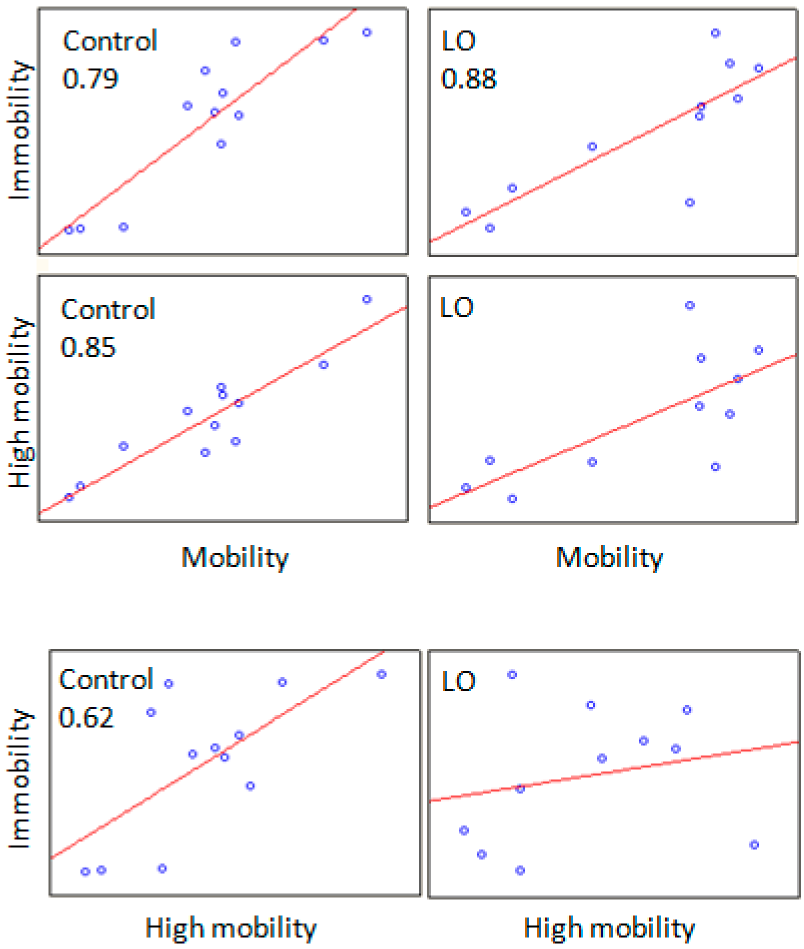

Supplement: Supplementary file 1 [file ijms-18-00560-s001.pdf]
